# Diagnostic, Prognostic, and Immunological Roles of FABP4 in Pancancer: A Bioinformatics Analysis

**DOI:** 10.1155/2022/3764914

**Published:** 2022-12-08

**Authors:** Jing Yang, Xiaojing Liu, Yueqin Shao, Hong Zhou, Lijun Pang, Wei Zhu

**Affiliations:** ^1^Oncology Center, The Affiliated Jiangsu Shengze Hospital of Nanjing Medical University, Wujiang, Jiangsu 215228, China; ^2^Department of Oncology, First Affiliated Hospital of Nanjing Medical University, Nanjing, Jiangsu 210029, China; ^3^Department of Radiotherapy, The Friendship Hospital of Ily Kazak Autonomous Prefecture, Ily, Xinjiang 835000, China

## Abstract

**Background:**

Fatty acid binding protein 4 (FABP4) is mainly involved in the regulation of systemic metabolism through various lipid signaling pathways. Metabolic reprogramming is one of the important factors in the development and progression of cancer. It has been recently reported that FABP4 is closely related to the development of cancer and may be involved in tumor invasion and metastasis.

**Methods:**

In this study, we explored the expression pattern of FABP4 in pancancer through TCGA and CPTAC. Using TCGA, Kaplan-Meier Plotter, and STRING databases, to explore its diagnostic and prognostic value, and function through GO/KEGG and GSEA. Then, using the TIMER2.0 database, we investigated the correlation between FABP4 expression and immune infiltration in cancers, especially stomach adenocarcinomas (STAD) and colorectal adenocarcinoma (COADREAD).

**Results:**

Compared with normal tissues, the expression of FABP4 in more than 10 tumor tissues was lower (*p* < 0.05). Through the receiver operating characteristic (ROC) curve, the diagnostic value was found higher in colorectal cancer, breast cancer, thyroid cancer, and lung cancer, with the area under the curve (AUC) > 0.9. Through the K-M curve, FABP4 was found to correlate to the prognosis of various cancers. The results of gastric cancer and colorectal cancer are consistent. The low-expression group has a better prognosis than the high-expression group, and the expression of FABP4 in the early T and N stages of gastrointestinal tumors is lower. FABP4 highly expressed gene set is mostly enriched in extracellular matrix degradation and cell adhesion functions. Gastrointestinal tumors with high expression of FABP4 may have more immunosuppressive effects on macrophages and have a worse prognosis.

**Conclusion:**

FABP4 can be used as a diagnostic and prognostic biomarker in pancancer, and its high expression in gastrointestinal tumors suggests poor prognosis. This may be correlated to the immune infiltration of macrophages and epithelial-mesenchymal transition.

## 1. Introduction

FABP4 has been reported as a lipid “chaperone” which regulates fatty acid metabolism and is involved in systemic metabolic regulation through multiple lipid signaling pathways. The FABP4, another name for adipocyte fatty acid-binding protein (A-FABP), is a fatty acid-binding protein, mainly expressed in adipocytes and macrophages. It has been studied to play an important role in cardiovascular and cerebrovascular diseases, and circulating FABP4 is a novel prognostic biomarker in patients with acute ischemic stroke [1]. Elevated plasma FABP4 concentrations were associated with a slightly higher risk of heart failure in American older adults in a prospective study of 4179 participants [2]. FABP4 plays an important role in macrophage inflammation and lipid metabolism in atherosclerosis [3], and serum FABP4 is strongly associated with cardiovascular disease mortality [4]. Therefore, the pathogenic cause may be related to endoplasmic reticulum stress [5] and inflammatory cytokines in macrophages [6].

Obesity may lead to an increased risk of cancer, and FABP4, as a member of the fatty acid-binding protein family, plays an important role in lipid regulation. FABP4 promotes the occurrence of obesity-related breast cancer and is a novel role in linking obesity and breast cancer risk [7]. The expression of FABP4 is upregulated or downregulated in a variety of cancers. FABP4 is highly expressed in breast-invasive lobular carcinomas [8], and the expression level of FABP4 protein in colorectal tumor tissue is higher than that in cancer-adjacent tissue [9]. The expression of FABP4 in hepatocellular carcinoma (HCC) tissues is lower than that in the adjacent tissue [10]. Meanwhile, FABP4 may be involved in tumor invasion and metastasis [11, 12], and adipose microenvironment regulation located in tumor-surrounding tissues plays a critical role in the regulation of the tumor microenvironment (TME) [13]. FABP4 has an important role in tumor proliferation, metastasis, and drug resistance, not only activating oncogenic signaling pathways but also remodeling the metabolism of tumor cells, providing energy requirements, and promoting tumor development. It may therefore be a promising target for cancer therapy [14]. Through the exploration of several databases, we tried to discuss the expression of FABP4 in pancancer, its value in diagnosis, survival, and its functional enrichment, and explore whether this process is related to immune infiltration.

## 2. Materials and Methods

### 2.1. Gene Expression Analysis

Download the RNAseq data in TPM (transcripts per million reads) format of TCGA (cancer tissue) and GTEx (healthy tissue) processed by Toil process [15] from the UCSC XENA platform (https://xenabrowser.net/datapages/). After log2 transformation, pancancer differential expression analysis was performed. The UALCAN web portal (http://ualcan.path.uab.edu/), provides a protein expression analysis option using data from Clinical Proteomic Tumor Analysis Consortium (CPTAC) Confirmatory/Discovery dataset. We obtained the difference in FABP4 protein expression in pancancer and normal tissues. And AUC was calculated by ROC curve to further judge the diagnostic value.

### 2.2. Survival Analysis

We analyzed the relationship between FABP4 expression and overall survival (OS) and recurrence-free survival (RFS), in stomach adenocarcinomas (STAD), liver hepatocellular carcinoma (LIHC), breast cancer (BRCA), and thyroid cancer (THCA) online by Kaplan-Meier Plotter (http://kmplot.com/analysis/). Hazard ratios (HRs) with 95% confidence intervals (CI) and log-rank *p* values were calculated. Then, use the survival data of colorectal adenocarcinoma (COADREAD) and lung adenocarcinoma and lung squamous carcinoma (LUADLUSC) in the TCGA database to supplement the missing colorectal cancer and lung cancer results in the Kaplan-Meier Plotter survival analysis, and calculate the overall survival (OS) and progression-free survival (PFI) by KM curve survival analysis.

### 2.3. Clinical Pathological Analysis

The relationship between FABP4 and clinicopathological features was carried out using the RNAseq data of the TCGA database STAD and COADREAD. We mainly explored the relationship between FABP4 expression and T and N stages of gastric and colorectal cancers, as well as its expression differences between CEA and H. pylori subgroups. *p* < 0.05 is considered a significant threshold.

### 2.4. Functional Enrichment and Gene Set Enrichment Analysis

The genes significantly correlated with FABP4 were confirmed using the online database cBioPortal (https://www.cbioportal.org/). Then, we downloaded the protein-protein interaction networks from String (https://string-db.org/) and selected 20 genes that interact with FABP4. The above genes were used to explore the role of FABP4 in biological function and signaling pathways. Using the R clusterProfiler package [16] performed Gene set enrichment analysis (GSEA) [17] of genes associated with differential expression of FABP4 in gastrointestinal tumors in the TCGA dataset.

### 2.5. Immune Infiltration Analysis

TIMER2.0 (http://timer.cistrome.org/) is a comprehensive resource for the systematic analysis of immune infiltrates across diverse cancer types. The abundance of tumor-infiltrating immune cells [18] was inferred from gene expression profiles. We analyzed the correlation between FABP4 expression and the abundance of immune infiltrating cells, including macrophage, mast cells, dendritic cells, and NK cells in gastrointestinal cancers by gene module. The correlation module generates scatter plots of FABP4 expression for specific cancer types and Spearman correlations and estimated statistical significance.

### 2.6. Statistical Analysis

Differences between groups were tested with the Mann–Whitney *U* test (Wilcoxon rank sum test). *p* < 0.05 is considered a significant threshold. The area under the ROC curve, AUC, represents the diagnostic accuracy. The closer the AUC is to 1, the better the diagnostic effect. When AUC is 0.5-0.7, it has low accuracy, when AUC is 0.7-0.9, it has certain accuracy, and when AUC is above 0.9, it has high accuracy. Log-rank test results of the Kaplan-Meier plot suggest HR and *p* values. The association of gene expression with immune infiltration was assessed by Spearman correlation, with absolute values of: 0.00-0.19 “very weak”, 0.20-0.39 “weak”, 0.40-0.59 “moderate”, 0.60-0.79 “strong”, 0.80-1.0 “very strong”, and *p* value <0.05 was considered statistically significant.

## 3. Results

### 3.1. FABP4 Expression in Human Cancers

**Figure 1:**
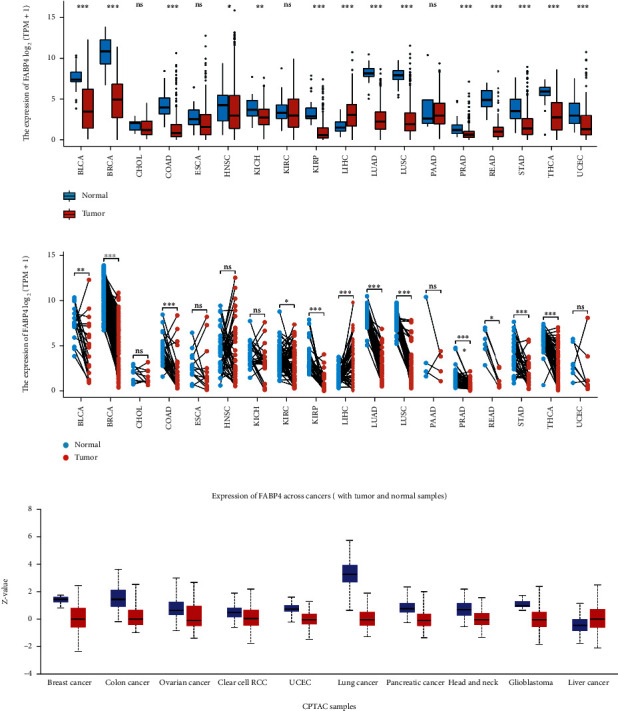
FABP4 expression in pancancer. (a) Differential expression analysis of FABP4 between unpaired cancer tissues and adjacent normal tissues in TCGA and GETx data. (b) Differential expression analysis between paired cancer tissues and adjacent normal tissues. (c) Expression of FABP4 protein in 9 kinds of tumors in CPTAC database. ns, *p* ≥ 0.05; ^∗^*p* < 0.05; ^∗∗^*p* < 0.01; ^∗∗∗^*p* < 0.001.

We analyzed the RNA expression levels of FABP4 in different tumor and normal tissues using the TCGA and GETx databases. The results showed that compared with normal tissues, the expression of FABP4 in the following tumor groups was lower than that in normal tissues, bladder cancer (BLCA), breast cancer (BRCA), colon adenocarcinoma (COAD), kidney renal papillary cell carcinoma (KIRP), lung adenocarcinoma (LUAD), lung squamous carcinoma (LUSC), prostate adenocarcinoma (PRAD), stomach adenocarcinomas (STAD), thyroid cancer (THCA) (*p* < 0.001), kidney renal clear cell carcinoma (KIRC), and rectal adenocarcinoma (READ) (*p* < 0.05), the differences were statistically significant. However, the difference was not statistically significant in the following tumor, cholangiocarcinoma (CHOL), esophageal carcinoma (ESCA), head and neck squamous cell carcinoma (HNSC), and pancreatic adenocarcinoma (PAAD) (*p* > 0.05). And the expression level in the liver hepatocellular carcinoma (LIHC) is higher than that of normal tissue (*p* < 0.001) (Figures 1(a) and 1(b)). The protein expression levels in different tumor and normal tissues were analyzed using the proteomic database CPTAC. The expression of FABP4 protein in 9 kinds of tumors including breast cancer, lung cancer, and colon cancer was lower than that in normal tissues, and there was statistical significance (Figure 1(c)).

### 3.2. Diagnostic Value of the Expression in Pancancer

**Figure 2:**
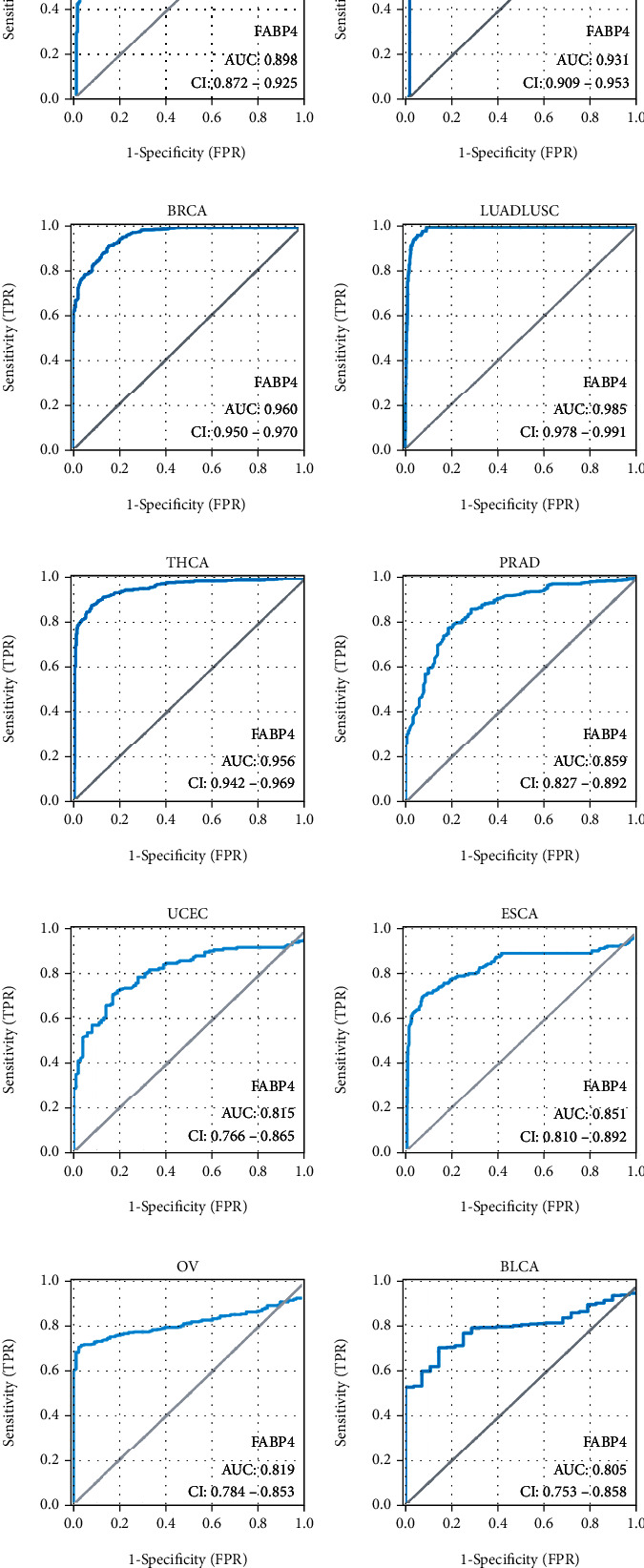
ROC curve analysis on the FABP4 gene expression in ten types of cancer. (a–j) The ROC curves of FABP4 in STAD, COADREAD, BRCA, LUADLUSC, THCA, PRAD, UCEC, ESCA, OV, and BLCA, respectively. STAD: Stomach Adenocarcinoma, COADREAD: Colon Adenocarcinoma and Rectum Adenocarcinoma, BRCA: Breast Invasive Carcinoma, LUADLUSC: Lung Adenocarcinoma and Lung Squamous Cell Carcinoma, THCA: Thyroid Carcinoma, PRAD: Prostate Adenocarcinoma, UCEC: Uterine Corpus Endometrial Carcinoma, ESCA: Esophageal Carcinoma, OV: Ovarian Serous Cystadenocarcinoma, BLCA: Bladder Urothelial Carcinoma.

We performed ROC curve analysis on the FABP4 gene expression data to assess the diagnostic value. The area under the curve (AUC) of FABP4 is 0.931 for colorectal cancer, 0.960 for breast cancer, 0.985 for lung cancer, 0.956 for thyroid cancer, and 0.898 for gastric cancer, which has a high diagnostic value. This value is 0.859 for prostate cancer, 0.851 for esophageal cancer, 0.815 for endometrial cancer, 0.819 for ovarian cancer, and 0.805 for bladder cancer, which also has a certain diagnostic value (Figure 2).

### 3.3. Prognostic Value of FABP4 in TCGA and Kaplan-Meier Plotter

**Figure 3:**
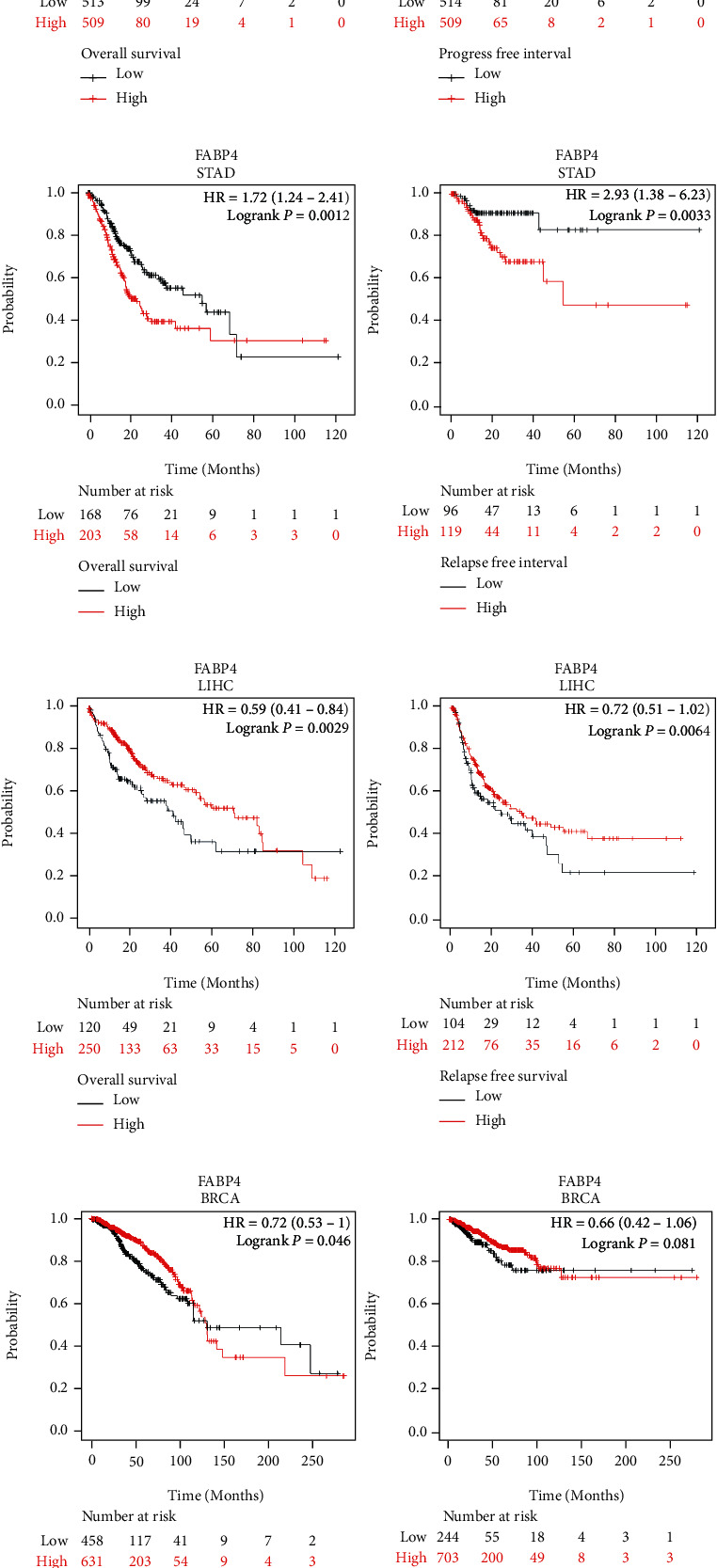
Kaplan-Meier survival curves comparing the high and low expression of FABP4 in different types of cancer in Kaplan-Meier Plotter. (a, b) OS and PFI of COADREAD and LUADLUSC. (c–f) OS and RFS of STAD, LIHC, BRCA, THCA, respectively. OS, overall survival; RFS, relapse-free survival; PFI, progress-free interval; COADREAD, Colon Adenocarcinoma and Rectum Adenocarcinoma; LUADLUSC, Lung Adenocarcinoma and Lung Squamous Cell Carcinoma; STAD, Stomach Adenocarcinoma; LIHC, Liver hepatocellular carcinoma; BRCA, Breast Invasive Carcinoma; THCA, Thyroid Carcinoma.

The online tool Kaplan-Meier Plotter was used to verify the association between FABP4 expression and prognosis in various cancers. The low-expression group of FABP4 in STAD had a better prognosis (OS: HR = 1.72, log-rank *p* = 0.0012, RFS: HR = 2.93, log-rank *p* = 0.0033) (Figure 3(c)). The other cancer types OS: LIHC (HR = 0.59, log-rank *p* = 0.0029) (Figure 3(d)), BRCA (HR = 0.72, log-rank *p* = 0.046) (Figure 3(e)), THCA (HR = 3.88, log-rank *p* = 0.023) (Figure 3(f)), RFS (relapse-free survival): LIHC HR = 0.72, log-rank *p* = 0.064) (Figure 3(d)), BRCA (HR = 0.66, log-rank *p* = 0.081) (Figure 3(e)), THCA (HR = 0.32, log-rank *p* = 0.0096) (Figure 3(f)). The TCGA database was used to supplement the prognostic results of COADREAD and LUADLUSC. The OS were: COADREAD (HR = 1.73, log-rank *p* = 0.002) (Figure 3(a)), LUADLUSC (HR = 1.00, log-rank *p* = 0.977) (Figure 3(b)), and PFI (progression-free interval): COADREAD (HR = 1.44, log-rank *p* = 0.02), LUADLUSC (HR = 0.98, log-rank *p* = 0.819). In addition, we also show the OS and RFS of LUSC, UCEC, CESC, and KIRC by Kaplan-Meier Plotter (*p* < 0.05) (Supplementary Figures 1(A)–1(D)).

### 3.4. Correlation Analysis of FABP4 Expression and Clinicopathological Features

**Figure 4:**
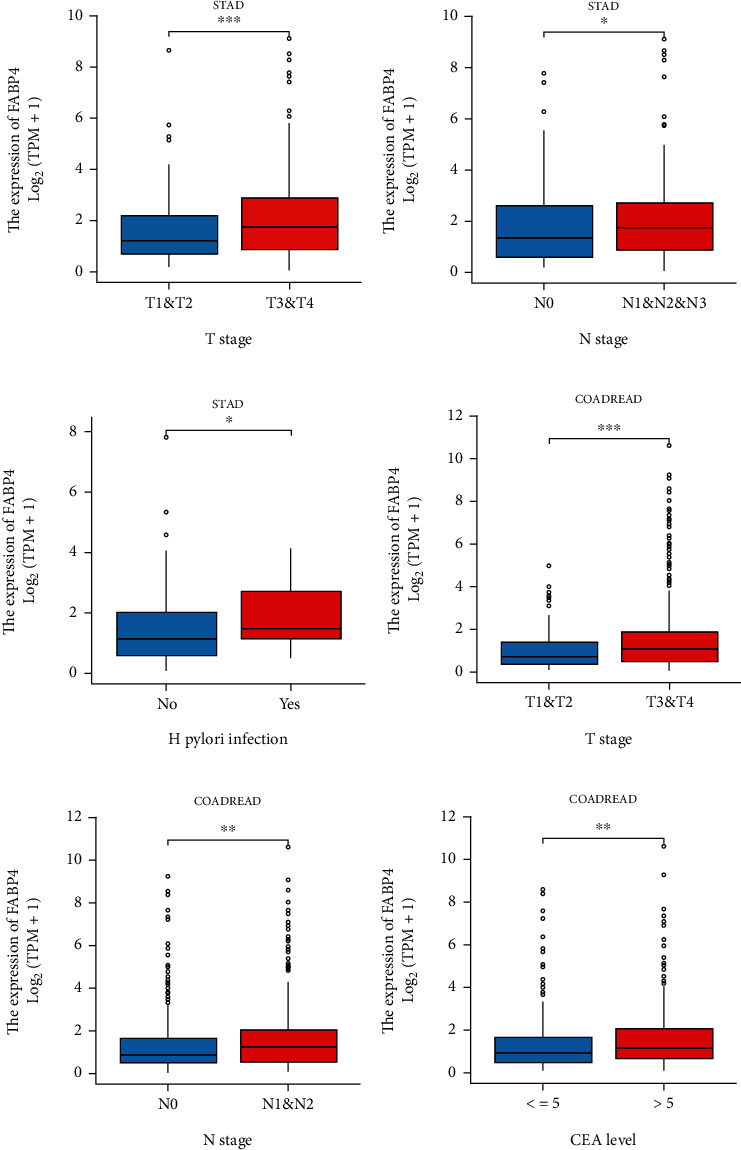
Association of FABP4 expression with clinical parameters. (a) T stage of STAD; (b) N stage of STAD; (c) Helicobacter pylori infection in STAD; (d) T stage of COADREAD; (e) N stage of COADREAD; (f) CEA level in COADREAD.

We analyzed the different clinicopathological features of FABP4 expression in patients with gastric and colorectal cancer. In both STAD and COADREAD, the expression level of FABP4 in the T3 and T4 stages is higher than that in T1 and T2 stages, and the difference is statistically significant (Figures 4(a) and 4(d)). In STAD, FABP4's expression in N+ (N1, N2, and N3) stage was higher than in the N0 stage (Figure 4(b)). A similar result was presented in COADREAD (Figure 4(e)). In colorectal cancer, the expression of FABP4 was significantly increased in the high CEA group (>5 ng/ml) compared with the low CEA group (<=5 ng/ml) (Figure 4(f)). FABP4 expression in gastric cancer is also associated with Helicobacter pylori infection (Figure 4(c)).

### 3.5. Functional Enrichment and GSEA

**Figure 5:**
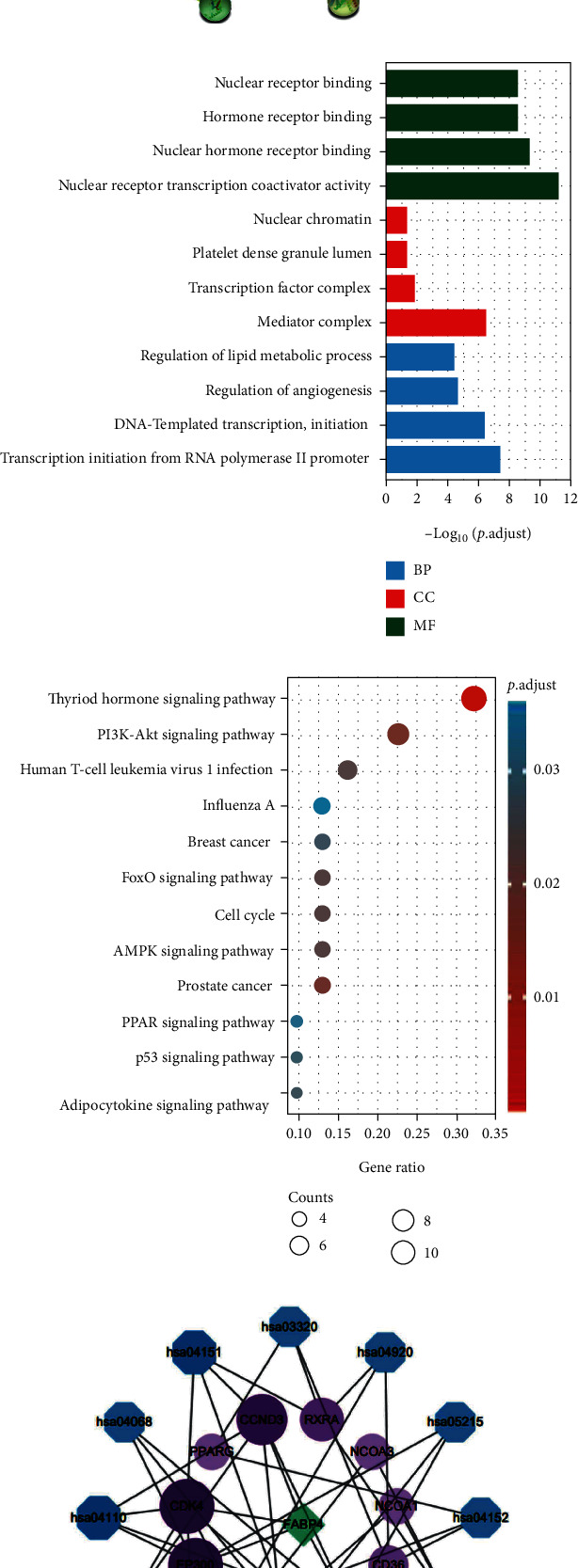
Gene Ontology and Kyoto Encyclopedia of Genes and Genomes pathway enrichment analyses. (a) 20 proteins interacting with FABP4 from the STRING database. (b) Column chart of GO enrichment analysis. (c) Bubble chart of KEGG pathway enrichment analysis. (d) FABP4, correlated genes, and pathway network diagram.

Functional interactions between proteins are essential for malignant tumors molecular mechanism and metabolism. To identify critical biochemical pathways, the STRING program was used to visualize protein-protein interactions and analyze enrichment. Protein-protein interaction subnetwork including 20 genes was extracted from the STRING database, while the minimum required interaction score: 0.7 (Figure 5(a)). Top 5 predicted functional partners: CREBBP, MED1, EP300, PPARG, and THRAP3.

The cBioPortal database was used to further confirm the 50 genes significantly correlated to FABP4, and the top 5 main related genes were as follows: CD36, MYCT1, EBF2, CD300LG, and AOC3. The above genes were successfully converted into 70 Entrez IDs (conversion ratios 100%). Under the conditions of p.adj < 0.05 and qvalue < 0.2 for GO and KEGG analyses, there were 234 BP, 4 CC, 32 MF, and 23 KEGG. GO analysis was visualized with a column chart (Figure 5(b)), and KEGG analysis was shown in a bubble chart (Figure 5(c)). The genes enriched in the KEGG analysis, FABP4, and the pathway was constructed into a network diagram (Figure 5(d)), the genes correlated to FABP4 were more enriched in the Cell Cycle (hsa04110), and the PI3K-Akt signaling pathway (hsa04151). The genes enriched in the KEGG analysis are shown in Supplementary Table 1.

**Figure 6:**
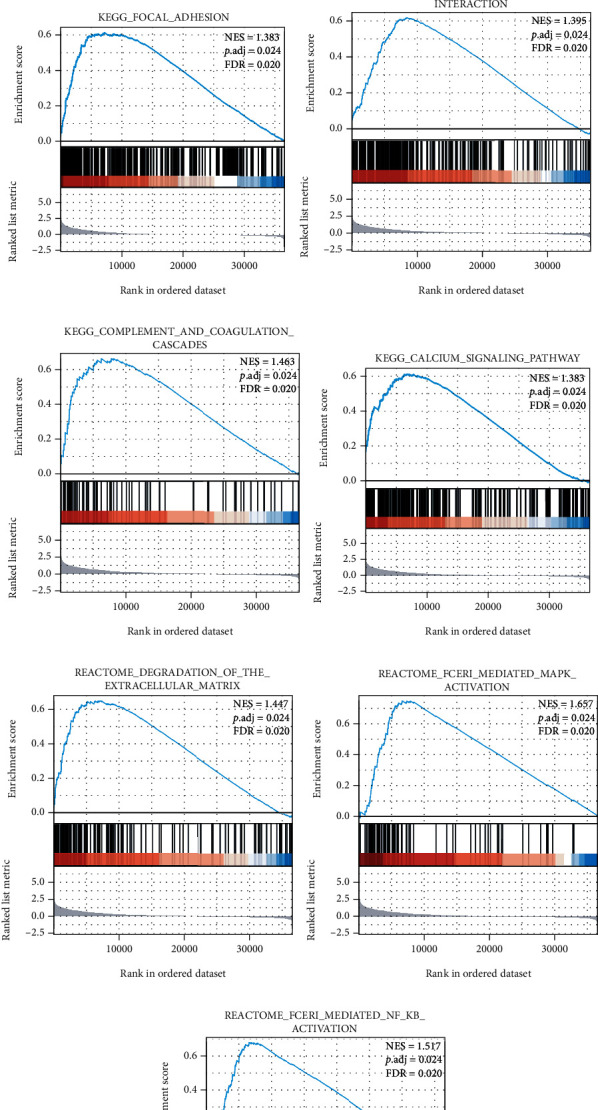
The gene set enrichment analysis result showed the most relevant enrichment pathway.

FABP4 differentially expressed gene analysis in gastric and colorectal cancer was performed using R DESeq2 [19] through the TCGA database, and GSEA was used to identify FABP4 correlated signaling pathways. We selected 9 datasets that satisfy FDR (qvalue) < 0.25 and p.adjust < 0.05 with higher Normalized Enrichment Score (NES) values, more of which were related to extracellular matrix adhesion function (Figure 6).

### 3.6. Analysis of Immune Cell Infiltration

**Figure 7:**
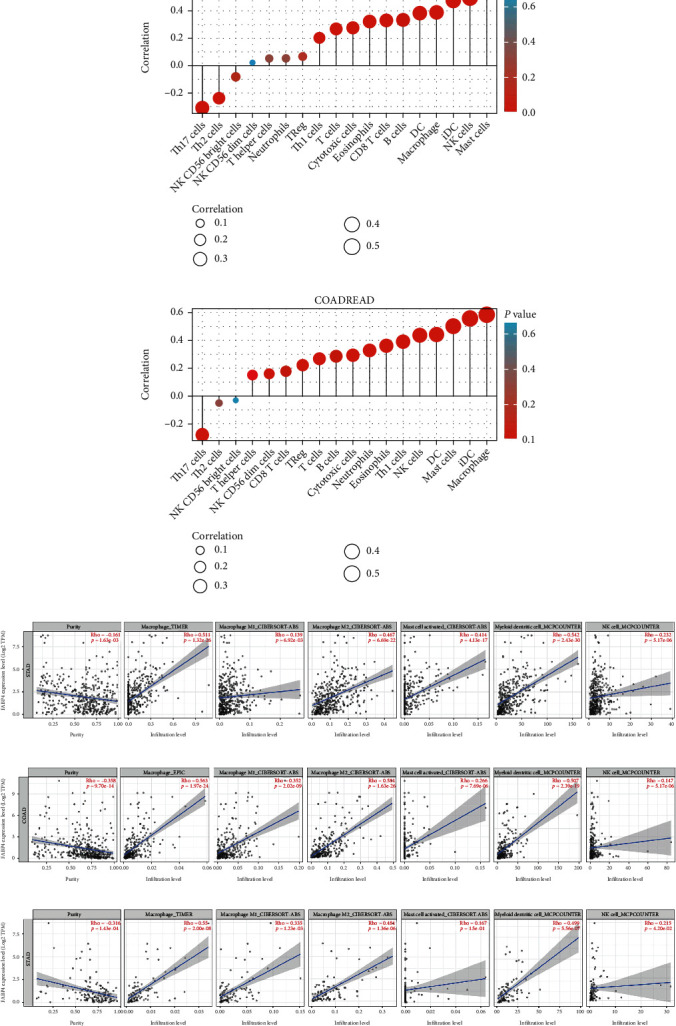
Lollipop-diagram for correlation between FABP4 and immune cell infiltration in stomach cancer (a) and colorectal cancer (b). Correlation of FABP4 expression with macrophage, mask cell, dendritic cell, NK cell in STAD (c), COAD (d), READ (e).

The correlation between FABP4 and immune cells was calculated by Spearman correlation analysis. The immune cells and correlation coefficients with positive correlation with gastric cancer from high to low were: mast cells (0.569), NK cells (0.494), iDC (0.475), macrophages (0.388), and DC (0.384) (Figure 7(a)). The positive correlation with colorectal cancer was macrophages (correlation coefficient 0.585), iDC (0.558), mast cells (0.503), DC (0.442), and NK cells (0.437), respectively (Figure 7(b)). The differences in the enrichment of immune cells with different levels of FABP4 expression were analyzed through the TCGA database, results were shown in Supplementary Figures 2(A) and 2(B).

We analyzed the correlation between FABP4 expression and the abundance of immune infiltration (including macrophage, mask cell, dendritic cell, and NK cell) in gastrointestinal cancers by gene module, scatter plots were formed to visualize the correlation of their expression with the level of immune cell infiltration above gastrointestinal cancers (Figures 7(c), 7(d), and 7(e)). We found that FABP4 expression in gastrointestinal tumors was more correlated with M2 macrophages and DC cells. The Kaplan-Meier curves of macrophage immune infiltration and gastric cancer were further displayed by the Outcome module. We found that low expression FABP4 may be a better prognosis, this is consistent with our previous Kaplan-Meier Plotter prognostic analysis results, and the prognosis of the high macrophages group is worse (*p* = 0.04) (Supplementary Figure 3). It indicated that macrophage infiltration may be one of the factors of poor prognosis in gastric cancer.

## 4. Discussion

FABP4 is a fatty acid-binding protein, which has been related to the occurrence and progression of chronic metabolic diseases and may directly affect drug discovery in metabolic diseases [20]. When cancer cells are deprived of nutrients, lipids in the surrounding microenvironment provide their energy-metabolic requirements. These reprogramming activities are now considered hallmarks of cancer [21]. Therefore, the relationship between lipid metabolism and cancer was increasingly close. The role of FABP4 in cancer has yet to be discovered, so in this article, we explored the potential role and mechanism of FABP4 in pancancer through bioinformatics analysis.

First, we found that FABP4 was low-expressed in more than ten cancers through pancancer analysis of the TCGA database and then confirmed its protein expression in the CPTAC database to be lower than that in normal tissues. The same findings were also made in previous studies. In breast cancer metastasis and recurrence subsets, the expression of the FABP4 protein is downregulated, while the expression of other proteins is upregulated [22]. The expression of FABP4 in hepatocellular carcinoma tissues was lower than that in the corresponding paracancerous tissues [10]. Decreased expression of FABP4 was detected in colorectal cancer patients and colorectal cancer cells [23]. The analysis of mRNA and protein expression in the experiment confirmed that the expression of FABP4 in endometrial cancer tissue was lower than that in normal endometrial tissue [24]. Nonetheless, the investigators' findings are inconsistent, FABP4 is highly expressed in breast cancer and may play a key role in metastasis and stromal cell interactions [8, 25]. Tumors that are prone to grow and metastasize are promoted in an adipocyte-rich microenvironment [12]. FABP4 is involved in the regulation of metastasis-related pathways and affects the metabolic pathways of ovarian cancer cells, which can significantly increase the metastatic potential of cancer cells [11, 26]. Therefore, we considered that FABP4 is low-expressed in various malignant tumors compared with normal tissues, but it may be upregulated during tumor development and metastasis and affect various metabolic pathways to promote cancer metastasis.

Then, in ROC analysis, we calculated AUC and found that FABP4 has a high value in the diagnosis of gastric cancer, colorectal cancer, breast cancer, and thyroid cancer. In this regard, FABP4 was previously found to be a potential biomarker for the diagnosis of colorectal cancer in Chinese patients [9]. FABP4 is mainly expressed in adipocytes and macrophages and can act as a functional marker of tumour-associated macrophages in primary tumours, and is a key molecular sensor regulating the differentiation and function of primary tumour macrophages, leading to the progression of ductal carcinoma in situ (DCIS) of the breast to progression of invasive disease [7]. Adipocytes supply fatty acids in the tumour microenvironment to provide metabolic energy and a key molecule in this process, FABP4, can influence *β*-oxidation in breast cancer cells, which in combination with free fatty acids (FFA) supports and promotes cancer cell survival and growth [27]. FABP4 is thought to play an important role in insulin resistance in T2DM [28]. Thus, FABP4 has also been found to be associated with insulin resistance in the survival prognosis of THCA patients [29]. Inhibition of FABP4 expression increases lipid droplet catabolism, which may help to reduce tumour growth in lung cancer patients by increasing endogenous ROS levels [30]. We considered that FABP4 may be involved in regulation of tumour-associated macrophage function, lipid droplet catabolism, fatty acid metabolism for energy supply and insulin resistance, and may potentially influence tumour development and progression.

Next, the potential value of FABP4 in cancer prognosis was verified by the K-M curve. It was found that in gastric cancer and colorectal cancer, FABP4 was correlated with overall survival and progression-free survival, and it was also correlated with overall survival in LIHC, BRCA, and THCA. Previous studies have concluded that high FABP4 expression is associated with poor overall survival in patients with liver cancer [10]. Our analysis has the similar result. In hepatocellular carcinoma, due to the inconsistent survival results, we further performed a GSEA analysis of FABP4 differences in TCGA-LIHC data and found that the functional enrichment of low expression gene sets in hepatocellular carcinoma was more significant in extracellular matrix regulation and epithelial-mesenchymal transition (EMT) processes (Supplementary Figure 4). Some investigators found that FABP4 inhibited HCC cell proliferation and invasion in vitro while leading to the inhibition of tumor growth and reduction in tumor size in vivo. They found that these were associated with altered expression of the epithelial-mesenchymal transition markers Snail and p-STAT3. Thus, low expression of FABP4 plays a key role in the proliferation and metastasis of HCC cells [10]. Conversely, it has also been shown that exogenous FABP4 stimulates HCC cell proliferation and migration through the activation of ERK and/or JNK signaling pathways [31]. Moreover, activation of JNK signaling may enhance the transactivation of TGF *β*1 and exacerbate liver fibrosis [32]. Thus, downstream effectors of related pathways remain to be discovered, and FABP4 may play different roles in the metastasis and/or progression of hepatocellular carcinoma. Meanwhile, disease-free survival (DFS) was found to be significantly correlated with FABP4 expression in breast cancer (*p* = 0.049) [33]. Increased FABP4 expression is associated with worse OS and lymph node metastasis, which may be a potential and promising biomarker to assess lymph node status and survival, and may also be a new therapeutic target for patients with node-positive cervical cancer [34, 35]. Similarly, high expression of FABP4 is considered to be associated with poor prognosis in breast, prostate, ovarian, and pancreatic cancers [36–38].

In our study, it was also found that gastric and colorectal cancers with high FABP4 expression had a worse prognosis. Therefore, we further explored its relationship with immune infiltrating cells in gastric and colorectal cancer and found that it was mainly related to macrophage infiltration, and in gastric cancer, macrophage infiltration was significantly correlated with prognosis. It has been identified in extensive experiments that FABP4 is predominantly expressed in adipocytes and macrophages. Inhibition of FABP4 in wound macrophages reduces inflammatory cytokine expression, making FABP4 a potent regulatory target for excessive inflammation and wound repair in diabetic patients [6]. FABP4 plays an important role in macrophage inflammation and lipid metabolism in atherosclerosis [3]. So its role in cancer may also be associated with macrophages. Hao et al. reported that the expression of adipocyte/macrophage A-FABP in tumor-associated macrophages (TAMs) promoted breast cancer progression. Loss of A-FABP expression significantly reduced breast cancer growth and metastasis. Therefore, the antitumor effect of A-FABP might be mediated by TAM [36]. In addition, FABP is widely expressed in CD8^+^ Tissue-resident memory T cells in different organs, and FABP expression profiles are shared by different immune cells in the same microenvironment [39]. Therefore, it reflects that FABP may play an important role in the immune microenvironment.

According to the cBioPortal database, CD36 is the gene with the greatest correlation with FABP4. CD36 and FABP4 are concomitantly expressed, and their direct interaction regulates fatty acids. Inhibition of CD36 and FABP4 induces apoptosis in breast cancer cells and inhibits the growth of mouse xenografts [40]. In GO/KEGG analyses, we found indirect roles of FABP4 in the PI3K-Akt signaling pathway, prostate cancer, AMPK, and PPAR signaling pathways. The function of FABP4 is achieved by inhibiting the phosphorylation of the PI3K/Akt signaling pathway [24]. Exogenous FABP4 activates the PI3K/Akt pathway independent of binding to fatty acids [41]. The transcriptional activity of PPAR*γ* was deactivated by the oncogene, resulting in the induced expression of the lipolytic gene FABP4, which was accompanied by a reduction in lipid droplets and tumor growth. This suggests that FABP4 has tumor suppressor effects in lung and renal cell carcinoma [30]. FABP4 overexpression inhibits tumor growth through the activation of PPAR*γ* [23].

In the process of exploring the mechanism and function of FABP4 in cancer, many researchers have found that it is related to the EMT process. We also found that FABP4 was involved in the adhesion function of extracellular matrix molecules by GSEA analysis. FABP4 enhances the EMT process of colon and cervical cancer cells through the AKT pathway and promotes the migration, invasion, and cytoskeleton reorganization of colon cancer [42, 43] and cervical cancer cells [34, 44], indicating that FABP4 promotes metastasis. In addition, gene set enrichment analysis found that its function was also expressed in the MAPK and NF-*κ*B pathways. The MAPK pathway is located downstream of many growth factor receptors and belongs to the cell proliferation signaling pathway. It may become a new therapeutic target for colorectal cancer [45]. The regulatory role of MAPK signaling in metabolism is linked to obesity-related cancers [46]. In an in vitro study, FABP4 expression was found to play an important role in oral squamous cell carcinoma cell growth through the MAPK pathway [47]. The expression of FABP4 in macrophages is associated with poor prognosis, and it may inactivate the NF-*κ*B-IL1*α* pathway, thereby promoting the proliferation and migration of neuroblastoma cells [48]. In general, FABP4 may be correlated to pathways such as tumor cell proliferation, apoptosis, metabolism, and inflammatory responses.

Although we uncovered a potential mechanism of FABP4 in tumorigenesis and its predictive value in clinical outcomes, this study still has limitations. First, we could not define FABP4 as a protective or risk factor because of some conflicting results from different studies. Second, this study only performed bioinformatics analysis of FABP4 expression and patient survival in different databases and did not perform in vivo/in vitro experiments. Third, the number of healthy subjects used as controls differed significantly from the number of cancer patients when performing variance analysis and survival analysis, so additional studies were needed to keep the sample size balanced. Fourth, while multicenter studies in public databases complement single-center studies, retrospective studies have limitations, such as inconsistent interventions. To further validate the role of FABP4 in the oncology field, and the results derived from bioinformatics predictions, additional prospective studies are needed.

## 5. Conclusions

Taken together, our pancancer analysis revealed for the first time that the expression of FABP4 was statistically significant with clinical diagnosis and prognosis, pathological staging, immune infiltration. It may affect tumor invasion and metastasis through multiple metabolic pathways, macrophages in the TME and EMT. This contributes to a more comprehensive understanding of the role of FABP4 in tumorigenesis and the search for potential therapeutic targets.

## Data Availability

The datasets presented in this study can be found in online repositories. The data used to support the findings of this study are available from the corresponding author upon request.

## Supplementary Materials

Supplementary Materials Supplementary Table 1: genes enriched in KEGG analysis. Supplementary Figure 1: Kaplan-Meier survival curves comparing the high and low expression of FABP4 in LUSC (A), UCEC (B), CESC (C), and KIRC (D). LUSC, lung squamous carcinoma; UCEC, Uterine Corpus Endometrial Carcinoma; CESC, cervical squamous cell carcinoma; KIRC, kidney renal clear cell carcinoma; OS, overall survival; RFS, relapse-free survival. Supplementary Figure 2: differences of immune cell infiltration in stomach cancer (A) and colorectal cancer (B) in groups with low and high expression of FABP4. Supplementary Figure 3: prognostic correlation of macrophage infiltration and FABP4 expression in gastric cancer. Supplementary Figure 4: the gene set enrichment analysis result showed the most relevant enrichment pathway on the basis of single-gene differential analysis of FABP4 in hepatocellular carcinoma.
