# Supplementary material for: Diagnostic, Prognostic, and Immunological Roles of FABP4 in Pancancer: A Bioinformatics Analysis
Source: Comput Math Methods Med. 2022 Dec 8;2022:3764914. doi: 10.1155/2022/3764914 (PMC9754845; doi:10.1155/2022/3764914)
Supplement: Supplementary Materials — Supplementary Table 1: genes enriched in KEGG analysis. Supplementary Figure 1: Kaplan-Meier survival curves comparing the high and low expression of FABP4 in LUSC (A), UCEC (B), CESC (C), and KIRC (D). LUSC, lung squamous carcinoma; UCEC, Uterine Corpus Endometrial Carcinoma; CESC, cervical squamous cell carcinoma; KIRC, kidney renal clear cell carcinoma; OS, overall survival; RFS, relapse-free survival. Supplementary Figure 2: differences of immune cell infiltration in stomach cancer (A) and colorectal cancer (B) in groups with low and high expression of FABP4. Supplementary Figure 3: prognostic correlation of macrophage infiltration and FABP4 expression in gastric cancer. Supplementary Figure 4: the gene set enrichment analysis result showed the most relevant enrichment pathway on the basis of single-gene differential analysis of FABP4 in hepatocellular carcinoma. [file 3764914.f1.docx]

Supplementary Table 1: Genes enriched in KEGG analysis.

| pathway | gene ID |
| --- | --- |
| hsa04919 | CREBBP, EP300, MED1, RXRA, NCOA3, NCOA1, MED14, NCOA2, MED13L, MED30 |
| hsa04151 | CCND3, CDK4, KDR, PTEN, RXRA, TEK, VEGFC |
| hsa05215 | CREBBP, EP300, ERG, PTEN |
| hsa04152 | CD36, LIPE, PPARG, PPARGC1A |
| hsa04110 | CCND3, CDK4, CREBBP, EP300 |
| hsa05166 | CCND3, CDK4, CREBBP, EP300, PTEN |
| hsa04068 | CREBBP, S1PR1, EP300, PTEN |
| hsa04920 | CD36, RXRA, PPARGC1A |
| hsa05224 | CDK4, PTEN, NCOA3, NCOA1 |
| hsa04115 | CCND3, CDK4, PTEN |
| hsa03320 | CD36, PPARG, RXRA |
| hsa05164 | CCND3, CDK4, CREBBP, EP300 |


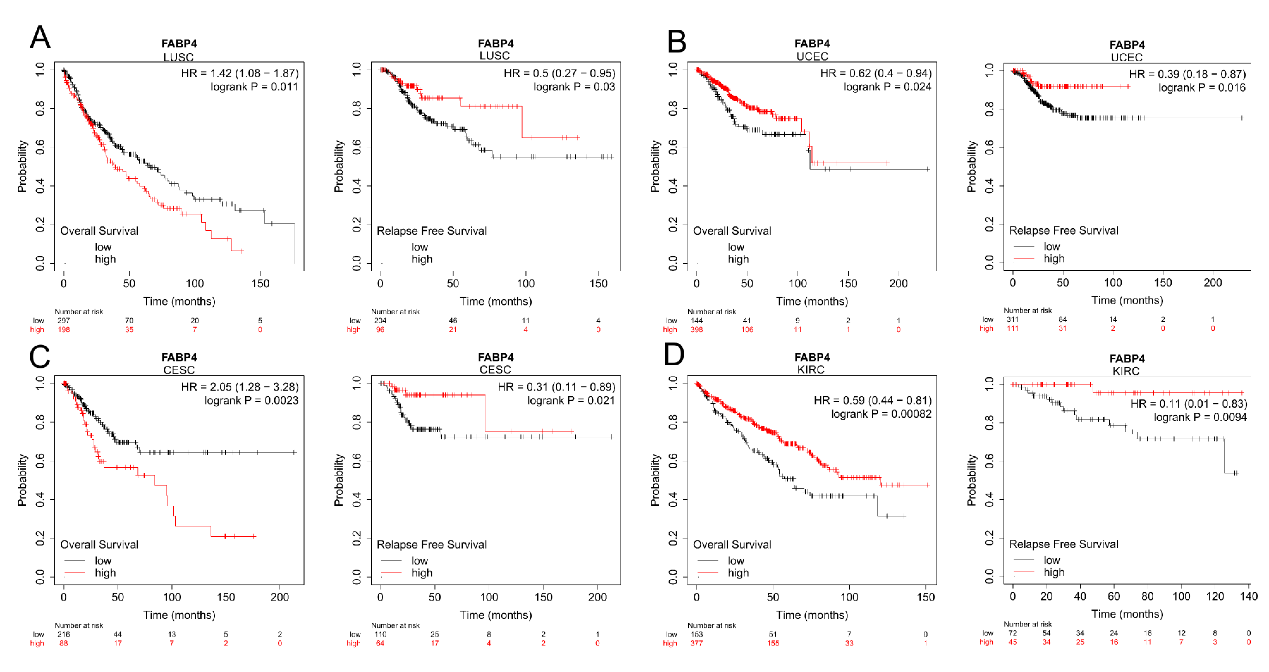


Supplementary Figure 1: Kaplan-Meier survival curves comparing the high and low expression of FABP4 in LUSC(A), UCEC(B), CESC(C), and KIRC(D).

LUSC, lung squamous carcinoma; UCEC, Uterine Corpus Endometrial Carcinoma; CESC, cervical squamous cell carcinoma; KIRC, kidney renal clear cell carcinoma; OS, overall survival; RFS, relapse-free survival.


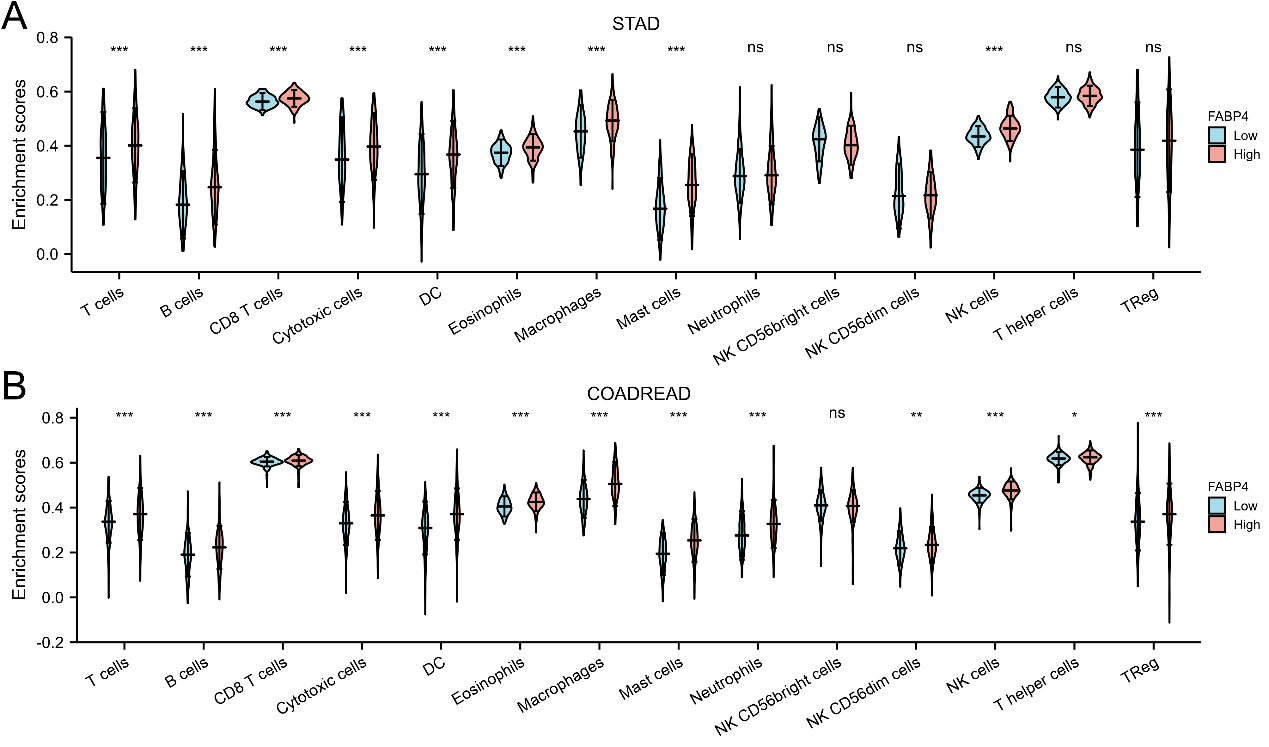


Supplementary Figure 2: Differences of immune cell infiltration in stomach cancer(A) and colorectal cancer(B) in groups with low and high expression of FABP4.


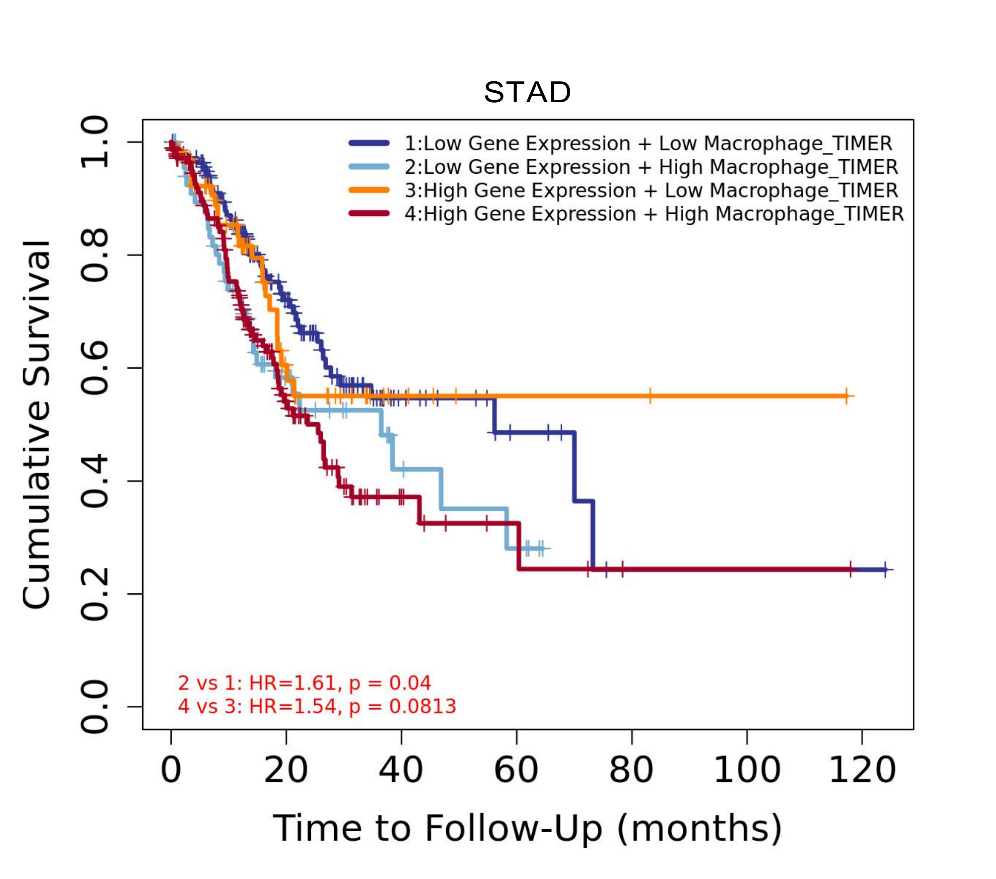


Supplementary Figure 3: Prognostic correlation of macrophage infiltration and FABP4 expression in gastric cancer.
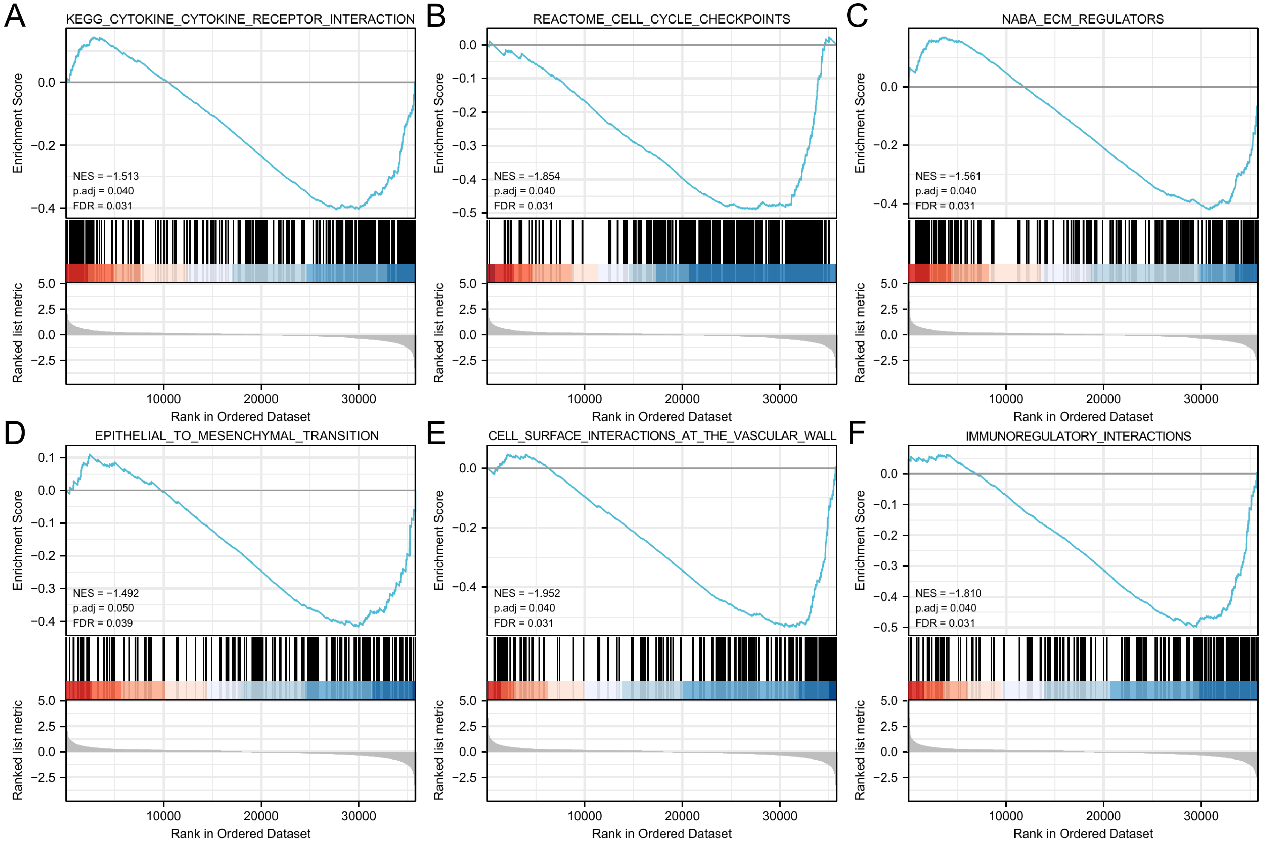


Supplementary Figure 4: The gene set enrichment analysis result showed the most relevant enrichment pathway on the basis of single-gene differential analysis of FABP4 in hepatocellular carcinoma.
